# Structural Mechanism for Regulation of Bcl-2 protein Noxa by phosphorylation

**DOI:** 10.1038/srep14557

**Published:** 2015-09-28

**Authors:** Christine B. Karim, L. Michel Espinoza-Fonseca, Zachary M. James, Eric A. Hanse, Jeffrey S. Gaynes, David D. Thomas, Ameeta Kelekar

**Affiliations:** 1 Department of Biochemistry, Molecular Biology and Biophysics, University of Minnesota, Minneapolis, MN 55455; 2 Department of Laboratory Medicine and Pathology and Masonic Cancer Center, University of Minnesota, Minneapolis, MN 55455; 3 College of Biological Sciences, University of Minnesota, Minneapolis, MN 55455.

## Abstract

We showed previously that phosphorylation of Noxa, a 54-residue Bcl-2 protein, at serine 13 (Ser13) inhibited its ability to promote apoptosis through interactions with canonical binding partner, Mcl-1. Using EPR spectroscopy, molecular dynamics (MD) simulations and binding assays, we offer evidence that a structural alteration caused by phosphorylation partially masks Noxa’s BH3 domain, inhibiting the Noxa-Mcl-1 interaction. EPR of unphosphorylated Noxa, with spin-labeled amino acid TOAC incorporated within the BH3 domain, revealed equilibrium between ordered and dynamically disordered states. Mcl-1 further restricted the ordered component for non-phosphorylated Noxa, but left the pSer13 Noxa profile unchanged. Microsecond MD simulations indicated that the BH3 domain of unphosphorylated Noxa is housed within a flexible loop connecting two antiparallel β-sheets, flanked by disordered N- and C-termini and Ser13 phosphorylation creates a network of salt-bridges that facilitate the interaction between the N-terminus and the BH3 domain. EPR showed that a spin label inserted near the N-terminus was weakly immobilized in unphosphorylated Noxa, consistent with a solvent-exposed helix/loop, but strongly constrained in pSer13 Noxa, indicating a more ordered peptide backbone, as predicted by MD simulations. Together these studies reveal a novel mechanism by which phosphorylation of a distal serine inhibits a pro-apoptotic BH3 domain and promotes cell survival.

BH3-only proteins of the Bcl-2 family share a single domain, the BH3 domain, through which they interact with multi-domain pro-survival family members, such as Bcl-2 or Mcl-1, and pro-apoptotic family members, such as Bax or Bak, to promote cell death^1^,^2^. The expression and activity of BH3-only proteins is, consequently, suppressed in healthy proliferating cells and in cancers by a variety of regulatory mechanisms. Some BH3-only proteins are constitutively expressed whereas others are expressed as a response to stress triggers, such as DNA damage or hypoxia^3^. A large body of evidence shows that constitutively active BH3-only proteins are maintained as inactive proteins until required, through post-translational mechanisms such as sequestration, tethering or phosphorylation (reviewed in^1^,^2^).

Human Noxa, the smallest of the known BH3-only proteins (54 residues), interacts with Bcl-2 protein Mcl-1_L_ via its BH3 domain to promote apoptosis. In most epithelial cells, the Noxa protein is induced in response to stress stimuli such as DNA damage and hypoxia^3^,^4^. Previously our group showed that this BH3 protein was constitutively expressed in leukemia cells but was phosphorylated at a single serine residue (Ser13) near its N terminus by a glucose-regulated kinase, and unable to activate apoptosis^5^. However, glucose withdrawal led to dephosphorylation of the serine and restored Noxa’s pro-apoptotic function. Constitutive expression and phosphorylation of Noxa also had a growth-promoting effect, imparting to cells increased dependence on glucose and diverting the sugar to biosynthetic metabolic pathways. Thus, a single modification had profound effects on cell fate, switching Noxa from a pro-apoptotic protein to one that promoted growth and survival. Although we hypothesized that phosphorylation was somehow preventing Noxa from interacting with its canonical binding partner, Mcl-1, it was unclear how a phosphoserine in the N-terminus could interfere with the binding interactions of the distally located BH3 domain (residues 29–35).

To address this question in the present study, we have examined the structural dynamics of human Noxa using electron paramagnetic resonance (EPR) spectroscopy, combined with molecular dynamics (MD) simulations and *in vitro* protein-protein interaction assays. EPR is a particularly powerful tool for detection of conformational states and structural changes in proteins and peptides in response to regulatory modifications, such as phosphorylation. Incorporation of the spin label amino acid TOAC (2,2,6,6-tetramethyl-piperidine-1-oxyl-4-amino-4-carboxylic acid) into the sequence of small proteins during their synthesis provides a compact probe that is rigidly coupled to the α-carbon and thus able to provide direct detection of peptide backbone dynamics by EPR spectroscopy^6^,^7^. EPR results were validated and complemented with microsecond all-atom MD simulations of both phosphorylated and unphosphorylated Noxa under physiological conditions. The MD simulations allowed us to resolve structural features of Noxa at a level of spatial and temporal detail not attainable through experimental approaches alone. Together, the studies described here reveal a simple, but novel, mechanism by which the phosphorylated Ser13 inhibits Noxa’s apoptotic function.

## Results

### EPR dynamics and Mcl-1 binding interactions of unphosphorylated and phosphorylated Noxa spin labeled in the BH3 domain

**Figure 1:**
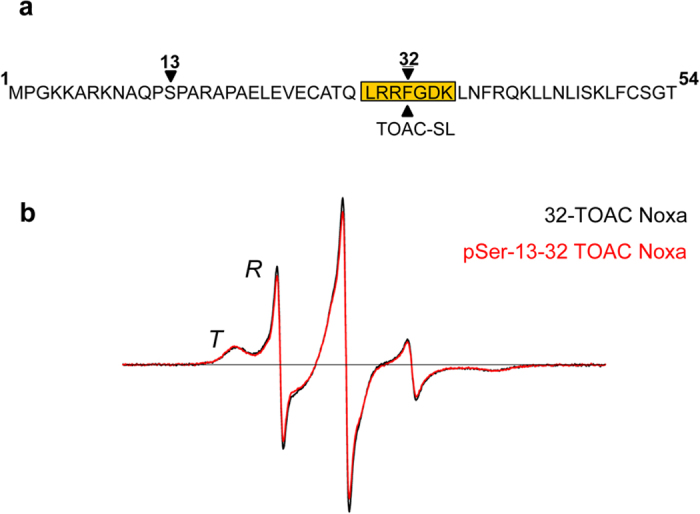
EPR dynamics of unphosphorylated and phosphorylated Noxa spin labeled in the BH3 domain. Human Noxa sequence with TOAC spin label inserted at position 32 in the BH3 domain (**a**). EPR spectra of Noxa in its unphosphorylated (black) and its Ser13 phosphorylated (red) state are shown overlaid (**b**). The two resolved conformations of the peptide backbone corresponding to an ordered (helical, *T* state) and dynamically disordered (unfolded, *R* state) conformation are indicated.

Previous studies from our group have demonstrated that BH3-only protein Noxa is constitutively expressed and phosphorylated in proliferating leukemic and primary human T cells. Phosphorylation renders Noxa unable to activate apoptosis through interaction with its canonical binding partner, Mcl-1^5^. To detect conformational and dynamic changes in Noxa that may result from phosphorylation at Ser13, the unnatural spin-labeled amino acid, TOAC, was incorporated into the BH3 domain of synthetic unphosphorylated and pSer 13 Noxa peptides at residue 32, in place of phenylalanine (Fig. 1a). The EPR spectrum of the spin-labeled BH3 domain (Fig. 1b) reports two conformations of the peptide backbone, corresponding to moderately restricted (*T*) and dynamically disordered (*R*) states. EPR spectra from unphosphorylated and pSer13-Noxa are virtually identical (Fig. 1b), indicating that phosphorylation does not significantly influence the *T-R* equilibrium.

**Figure 2:**
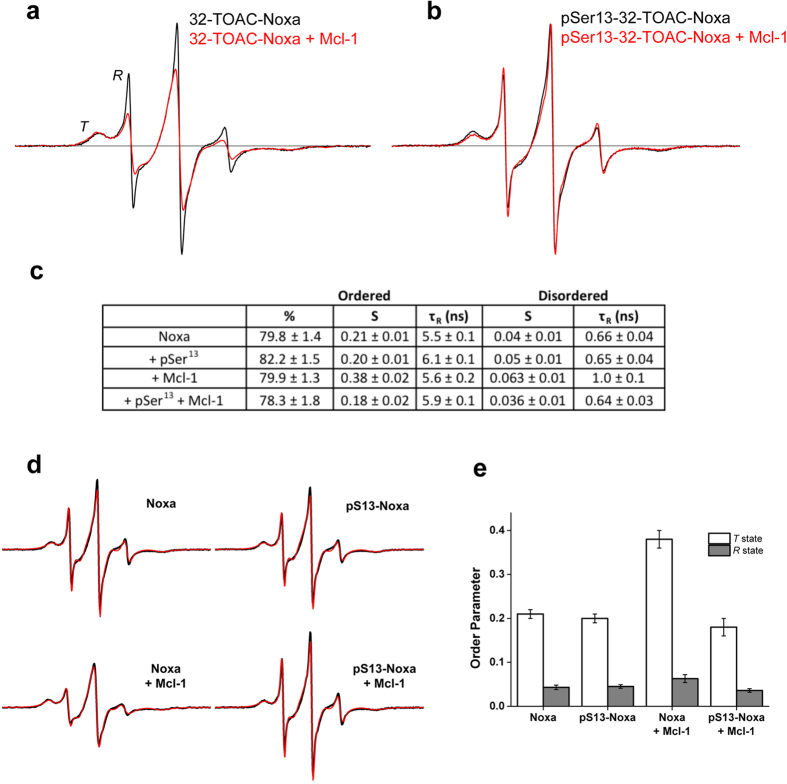
EPR dynamics and Mcl-1 binding interactions of unphosphorylated and phosphorylated 32-TOAC Noxa. EPR spectra of 32-TOAC-Noxa free in solution (black) and in the presence of excess Mcl-1 (red), in the absence (**a**) and presence (**b**) of phosphorylation at Ser13. All spectra show two components or structural states, a helically ordered state (*T*) and a dynamically disordered state (*R*). (**c)** Table showing results of EPR analysis, obtained by fitting each EPR spectrum using NLSL^8^, assuming two populations. Best fit parameters for the *T* state: τ_R _= 3.33 ± 0.08 ns, S = 0.56 ± 0.01. Best-fit parameters for the R state: τ_R _= 0.55 ± 0.13 ns, S < 0.05.S = order parameter, τ_R _= rotational correlation time. Uncertainties indicate the range of values that give acceptable fits. (**d)** EPR spectra (black) and best fits (red) of 32-TOAC-Noxa and pSer13–32-TOAC-NOXA in the presence and absence of Mcl-1, with the *T* state populations obtained from the fits. (**e**) Order parameters *S* of the *T* (white) and *R* (grey) states of Noxa ± Ser13 phosphorylation and/or Mcl-1. Error bars indicate the range of values that give acceptable fits, with the best fits illustrated in (**d**).

Next, we determined the EPR dynamics of Noxa and pSer13-Noxa in the presence of purified recombinant Mcl-1. Figure 2a shows that binding to Mcl-1 apparently decreases the intensity of the *R* state relative to that of the *T* state. In comparison, pSer13-Noxa reports essentially the same spectrum regardless of Mcl-1 addition (Fig. 2b). To quantify the *T* and *R*-state populations, and also to characterize the rotational dynamics of these states, we fit all acquired spectra using NLSL^8^, assuming the microscopic-order macroscopic-disorder (MOMD) model. Fitting results are given in the table in Fig. 2c with best fits to the spectra shown in Fig. 2d. We found the *T* and *R*-state populations to be largely invariant, while the order parameter *S* and correlation time τ_R_ of *T*-state Noxa increased dramatically upon Mcl-1 addition, indicating increased order and slower dynamics, respectively, consistent with binding (Fig. 2e). In contrast, the order parameter of *T*-state pSer13-Noxa did not change in the presence of Mcl-1, supporting the conclusion that phosphorylated Noxa does not bind Mcl-1. In all cases, the order parameter of the *R* state is <0.1, indicating essentially complete unfolding in that part of the sequence (residue 32).

### *In vitro* Noxa-Mcl-1 binding interactions

**Figure 3:**
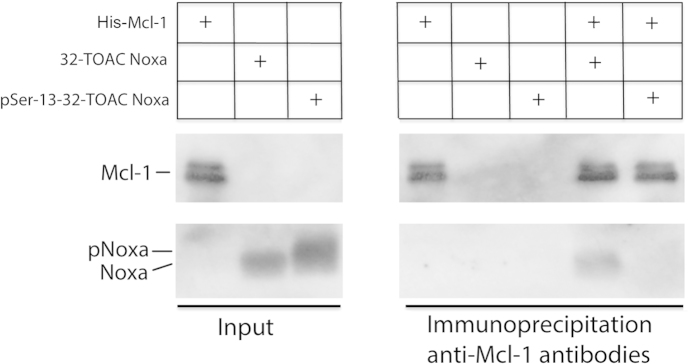
Mcl-1 binds to 32-TOAC Noxa, but not to pSer13–32-TOAC Noxa *in vitro*. Unphosphorylated or pSer13 32-TOAC Noxa peptides (20 ng) incubated for 1 hour with or without recombinant purified His-Mcl-1_L_ (120 ng) in Dignam Buffer were subjected to immunoprecipitation with 200 ng anti-Mcl-1 antibodies. Immunoprecipitated proteins were resolved by SDS-PAGE and western blotted for the presence of Noxa and Mcl-1. Input lanes are loaded with half the total amount of Noxa peptide and Mcl-1 used in the immunoprecipitation reactions.

To ascertain that TOAC-labeled Noxa was representative of the wild type protein and that incorporation of the TOAC label within the BH3 domain did not interfere with the Noxa- Mcl-1 interaction, we carried out direct binding assays *in vitro* using the TOAC labeled peptides and the recombinant purified His-tagged Mcl-1 used in the EPR studies. Noxa peptides that co-precipitated with Mcl-1 following immunoprecipitation with anti-Mcl-1 atibodies were detected by western blotting. Results (Fig. 3) show a binding interaction between 32-TOAC-Noxa and Mcl-1 but no interaction between Mcl-1 and pSer-13–32-TOAC Noxa, confirming that the TOAC spin label does not compromise the binding properties of the BH3 domain and further strengthening our hypothesis that Ser13 phosphorylation prevents the Noxa-Mcl-1 interaction.

### Structure and dynamics of Noxa and pSer-13-Noxa through Molecular Dynamics simulations

**Figure 4:**
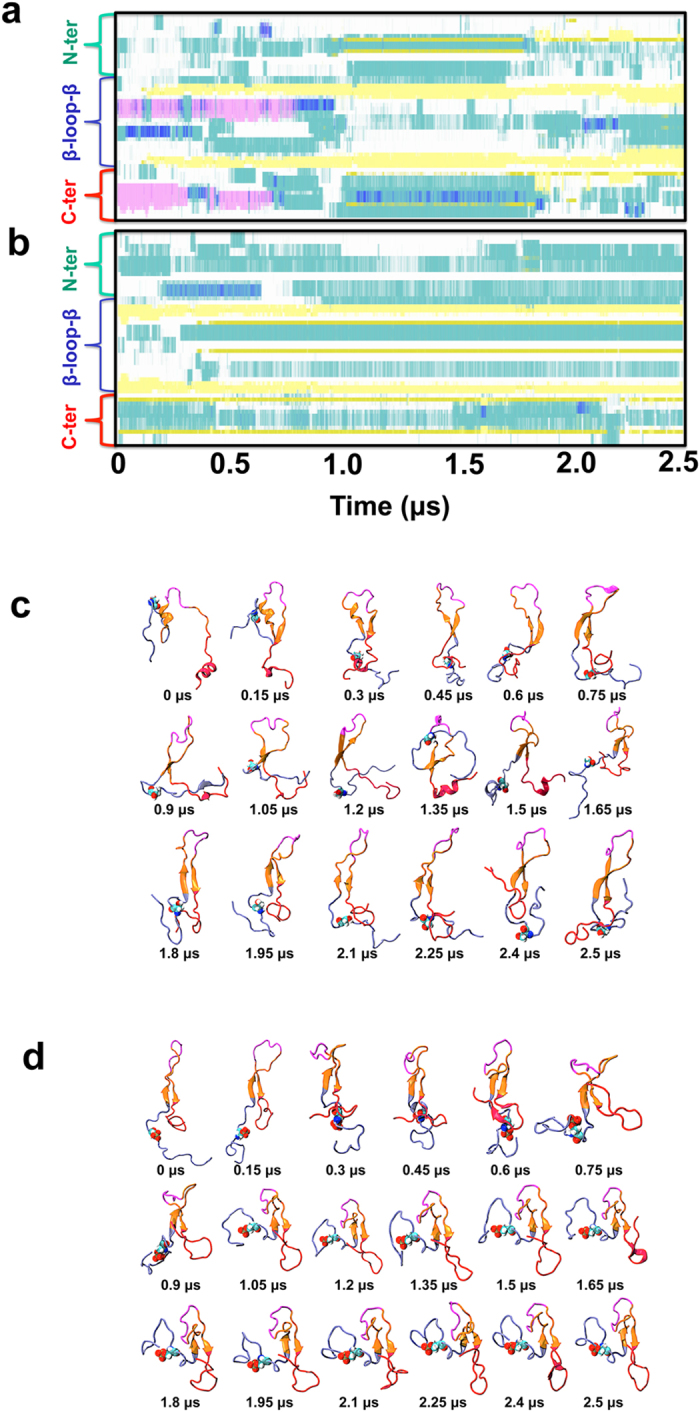
Time dependent evolution of the secondary structure of human Noxa. (**a**,**b**) Time-dependent changes in the secondary structure evolution of unphosphorylated Noxa and phosphorylated Noxa, respectively. Color codes: α-helix (pink), 3_10_-helix (blue); β-sheet (yellow); β-turn (cyan), isolated bridge (olive); random coil (white). (**c**,**d**) Structures of unphosphorylated Noxa and phosphorylated Noxa at different time intervals in the MD trajectory, respectively. Color scheme: N-domain, blue; C-terminus, red; β-loop-β motif, orange; BH3 domain, magenta.

**Figure 5:**
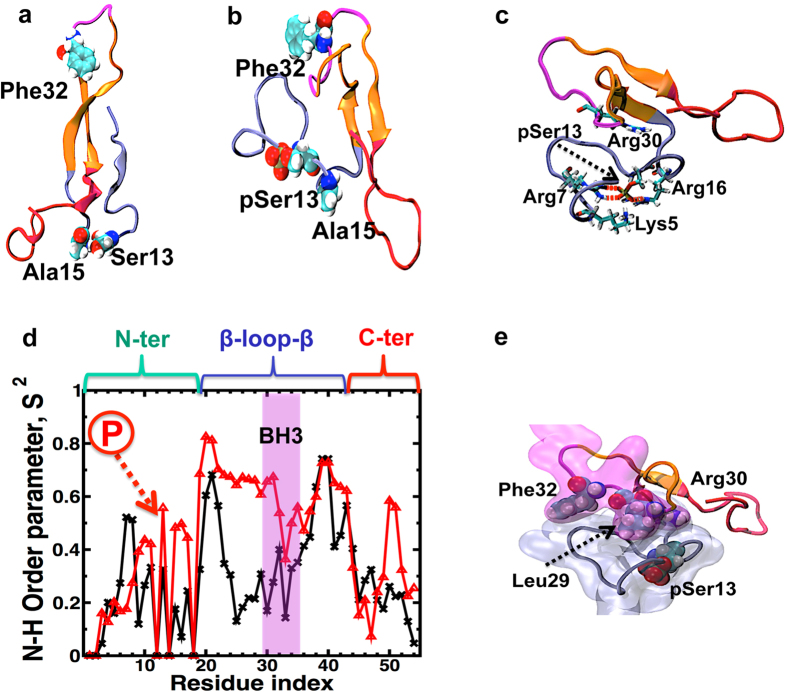
Structural dynamics of Noxa using MD simulations. (**a**) Ribbon representation of unphosphorylated Noxa at the end trajectory. (**b**) Structure of phosphorylated Noxa at t = 2.5 μs. TOAC labeling and phosphorylation sites are shown as van der Waals spheres. (**c**) Structure of phosphorylated Noxa showing the network of favorable electrostatic interactions centered round pSer13. Residues interacting via salt bridges (red dashed lines) and long-range electrostatic interactions are shown as sticks. (**d**) N-H peptide bond order parameters (S^2^) calculated for unphosphorylated (black line) and phosphorylated (red line) Noxa. The arrow indicates the position of the phosphorylation site (Ser13); the brackets indicate the location of the N-terminus, β-loop-β domain and C-terminus. The shaded magenta area shows the location of the BH3 domain. (**e**) Surface representation showing the interaction between the N-terminus and the BH3 domain. pSer13 and residues Leu29, Arg30 and Phe^32^ are shown as van der Waals spheres. Color scheme used in all cases: N-domain, blue; C-terminus, red; β-loop-β motif, orange; BH3 domain, magenta.

We performed two 2.5 μs MD simulations of unphosphorylated and phosphorylated Noxa, using microsecond molecular dynamics (MD) simulations. Analysis of the evolution of the secondary structure showed that Noxa possesses several unstructured regions (Fig. 4), indicating that Noxa is intrinsically disordered, in agreement with circular dichroism experiments^5^. However, MD simulations suggest that the structural ensemble of unphosphorylated Noxa does not consist of several dissimilar conformations that exchange rapidly in the microsecond time scale, as shown previously for other disordered proteins in solution^9^,^10^. Instead, Noxa folds into a relatively well-defined structure in solution, which consists of a β-loop-β motif (residues Ala19 to Lys41) flanked by two disordered segments at the N-terminus (residues Met1-Pro18) and the C-terminus (residues Asn44-Thr54), shown in Fig. 5a. We found that the BH3 domain (Leu29-Lys35) of unphosphorylated Noxa is located in the flexible loop of the β-loop-β motif (Fig. 5a, magenta). Analysis of the time-dependent changes of the secondary structure revealed that the initial structure of unphosphorylated Noxa, consisting of a random coil and two helical segments at the N- and C-terminus of the protein, folds into a stable β-loop-β motif in less than a microsecond (Fig. 4a,c, also described in^10^). Furthermore, we found that the β-loop-β motif of unphosphorylated Noxa remained stable for the rest of the simulation period.

To evaluate the effect of phosphorylation at Ser13 on the structural dynamics of Noxa, we used the structure at the end of the 2.5-μs trajectory of unphosphorylated Noxa shown in Fig. 5a. The structure of phosphorylated Noxa at the end of the simulation is shown in Fig. 5b. Analysis of the trajectory of phosphorylated Noxa showed that the β-loop-β structural motif remains stable during the entire simulation time (Fig. 4b,d). Time-dependent evolution of the secondary structure also showed that the N- terminus (which contains the phosphorylation site) and the BH3 domain remain unfolded in the microsecond time scale (Fig. 5b). These findings indicate that phosphorylation induces neither folding nor unfolding of Noxa in solution.

Closer inspection of the structure around the phosphorylated site of Noxa showed that phosphorylation forms a network of favorable electrostatic interactions with several basic residues of Noxa (Fig. 5c). Salt bridges were detected between the phosphate group of pSer13 and residues Lys5, Arg7 and Arg16 (Fig. 5c). In addition, long-range electrostatic interactions were detected between pSer13 and Arg30, a residue from within the BH3 domain. Such favorable electrostatic interactions were completely absent in unphosphorylated Noxa (Fig. 5a). In order to quantifiably evaluate the effect of the phosphorylation-induced favorable electrostatic interactions on the amplitude of Noxa backbone dynamics, we calculated the N-H peptide bond order parameters (S^2^) using the isotropic re-orientational eigenmode dynamics method^11^. The S^2^ values were calculated using structures obtained after 0.8 and 1 μs for unphosphorylated and phosphorylated Noxa, respectively. As expected from the structural analysis, S^2^ values revealed that the N- and C-termini as well as the loop connecting the β-sheets show a high degree of flexibility in solution (Fig. 5d, black line). Only a few residues located at the beginning and end of the β-loop-β motif were relatively rigid, indicating that these residues play a central role in the stability of this motif. We found that upon phosphorylation, there was no substantial change in the backbone dynamics of several residues, including TOAC-labeled Phe32, which supports the EPR data. However, we did find a substantial decrease in the amplitude of N-terminus backbone dynamics upon phosphorylation (Fig. 5d, red line), indicating a link between the salt-bridge network around the phosphorylation site and a decrease in structural flexibility of the N-terminus.

This ‘ordering’ effect of phosphorylation extends to the N-terminal segment of the β-loop-β motif, including residues Leu29 and Arg30 of the BH3 domain (Fig. 5d). The apparent decrease in backbone flexibility upon phosphorylation is not linked to the formation of well-defined secondary structure. Instead, the decrease in flexibility of the N-terminus and the favorable long-range electrostatic interaction between pSer13 and Arg30 facilitate the interaction of the N-terminus with the BH3 domain (Fig. 5b). The interaction between the N-terminus and the BH3 domain explains the apparent decrease in the backbone flexibility of the N-terminal region of the β-loop-β motif. This interaction also explains the inhibitory effect of phosphorylation, as residues that are important for recognition and binding to Mcl-1, such as Leu29, are “masked” by the N-terminus (Fig. 5e).

### EPR dynamics of 15-TOAC labeled Noxa reveal a more ordered backbone in the phosphorylated peptide

**Figure 6:**
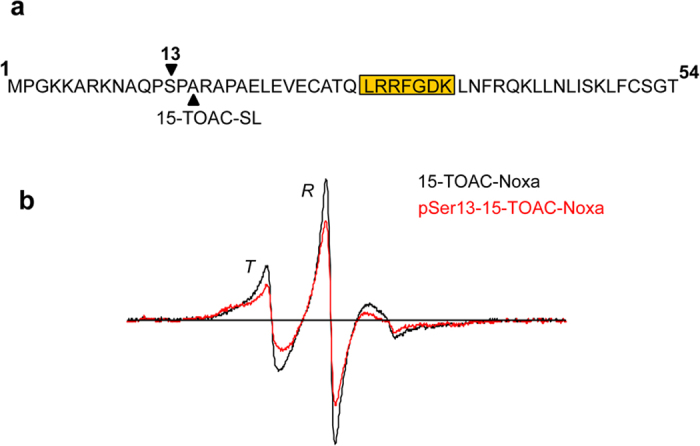
15-TOAC-Noxa EPR spectra indicate a more ordered backbone at the N-terminus of the phosphorylated peptide. Shown in the figure is an overlay of EPR spectra from unphosphorylated (black) and phosphorylated at Ser13 (red) Noxa with spin label inserted at position 15 into the sequence.

The simulations described above revealed a substantial decrease in the amplitude of N-terminus backbone dynamics (Fig. 5d, red line), suggesting a link between the salt-bridge network around the phosphorylation site and decreased structural flexibility in the N-terminus. To test this possibility, we examined the backbone dynamics of unphosphorylated and phosphorylated Noxa peptides with the TOAC spin label inserted at position 15, in place of alanine (Fig. 6a,b). The EPR spectrum in the absence of phosphorylation (black) indicates a weakly immobilized spin label, consistent with a locally unfolded peptide backbone, as in a solvent-exposed helix or loop. Phosphorylation of the peptide (red) produces a spectrum having broader features, indicating a more strongly immobilized spin label, and thus a more ordered peptide backbone. The phosphorylation-induced decrease in backbone dynamics observed by EPR, thus validates the prediction from the MD simulation. Taken together, these data suggest that although the BH3 domain of the phosphorylated protein is not structurally altered (Fig. 1), dynamic changes that occur in the vicinity of Ser13, following its phosphorylation, have the ability to affect the domain’s binding interactions.

## Discussion

**Figure 7:**
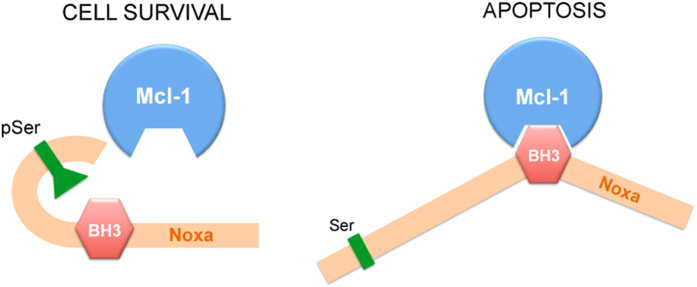
Regulation of the Noxa-Mcl-1 interaction via Serine 13 phosphorylation. Shown in the figure is a simplified cartoon demonstrating how the partial masking of the BH3 domain resulting from phosphorylation of Ser13 could inhibit the interaction of Noxa with Mcl-1.

In this study we have identified a structural change in the Noxa protein following phosphorylation of Ser13, that offers a simple explanation for the inability of the endogenous protein to bind Mcl-1 and, subsequently, to activate apoptosis in healthy proliferating cells (model in Fig. 7). MD simulations and EPR spectroscopy, combined with *in vitro* co-immunoprecipitations, have offered novel insights into a protein that has, to date, been resistant to structural resolution by NMR or X-ray crystallography. Published structural studies on Noxa have, for the most part, been restricted to the BH3 domain of either the murine or human protein in complex with purified recombinant Mcl-1^12^,^13^,^14^.

The MD simulations in our study suggested that human Noxa is intrinsically disordered but rather than assuming several conformations, as is the case with other disordered proteins^9^, Noxa appears to fold into a β-loop-β motif that encompasses the hydrophobic BH3 domain and is flanked by disordered N- and C-terminal segments^10^. While the amplitude of the backbone dynamics of the N-terminus showed a substantial decrease upon phosphorylation of Ser13, there was no detectable change in the backbone dynamics of the BH3 domain surrounding residue Phe32, suggesting that the decreased flexibility of the N-terminus was the result of the salt-bridge network around the phosphorylation site, rather than a well-defined secondary structure. The EPR data revealed a dynamic equilibrium between ordered and dynamically disordered conformations of the peptide backbone in both the unphosphorylated and pSer13-Noxa, and strongly support the simulation studies in detecting no structural changes around the TOAC label at residue 32 in the BH3 domain following Ser13 phosphorylation. These studies provide early structural insights into full length Noxa and open the possibility of further refinements of its structure in the future using a combination of EPR and simulation analyses.

The simulations also suggest that the reduced flexibility in the N-terminal region of the β-loop-β motif, combined with the favorable long-range electrostatic interaction between pSer13 and Arg30, would together result in partial masking of the BH3 domain, affecting Noxa’s ability to bind Mcl-1 and thus, its pro-apoptotic function. This prediction is clearly supported by the EPR data and by *in vitro* binding assays^5^. The addition of Mcl-1 decreased the intensity of the *R* (disordered) state but substantially increased the order parameter (*S*) of the *T*-state of unphosphorylated Noxa, consistent with binding. However, Mcl-1 very minimally destabilized the ordered conformation of pSer13-Noxa, supporting the lack of a binding interaction between the two proteins.

Human Noxa was, until recently, thought to be expressed primarily in response to stress triggers, such as DNA damage and hypoxia. The realization that pro-apoptotic Noxa is constitutively expressed in leukemia cells, and in proliferating primary cells of hematopoietic lineage, was the first step in the discovery of a post translational modification on the protein that prevented this BH3-only protein from assuming its pro-apoptotic role^5^. Not only is Noxa stably expressed in lymphocytic leukemias, it is also phosphorylated by the kinase Cdk5 to prevent it from functioning as a canonical death promoter in the leukemia cells. A recent study in Helicobacter pylori infected gastric epithelial cells by Rath *et al.*^15^ further underscores the importance of Ser13 phosphorylation on human Noxa. The authors show that a different kinase, JNK, phosphorylates Noxa, resulting in its retention in the cytoplasm. They also show that inhibition of the kinase promotes a mitochondrial Noxa-Mcl-1 interaction. Therefore, understanding the mechanism by which this modification controls Noxa function is critical and could lead to new therapeutic strategies to activate the endogenous protein either by kinase inhibition or by eliminating the putative salt bridges and electrostatic interactions that effectively mask the pro-apoptotic BH3 domain.

## Methods

### Recombinant protein purification

Wild type human Mcl-1 (Ct∆24), cloned into the NdeI/XbaI sites of pET15b, was a gift from Jason O’Neill (Fred Hutchinson Cancer Research Center). pET15b-Mcl-1 (N-terminus fused 6× His-tagged Mcl-1) was transformed into BL21 (DE3) pLysS *E. coli* (Novagen). Mcl-1 protein expression was induced for 16 h using the overnight auto-induction system (Novagen). Polyhistidine tagged Mcl-1 was purified by immobilized metal affinity chromatography from extracts prepared with bacterial protein extraction buffer (Pierce). Briefly, protein extracts were loaded onto a nickel resin packed column (Roche), washed, and bound proteins eluted in elution buffer (200 mM imidazole in Tris-buffered saline). Eluted proteins were resolved by SDS-PAGE and efficacy of purification was assessed by Coomassie staining and immunoblotting for Mcl-1. Purified Mcl-1 was concentrated and dialyzed into Tris-buffered saline (TBS).

### Peptide Synthesis

Noxa was synthesized as reported previously^5^. Fluorenylmethyloxycarbonyl (Fmoc) methodology was used for inserting the spin label probe paramagnetic amino acid TOAC in the protein for investigation by EPR spectroscopy^6^,^16^. The protocol for Fmoc-TOAC-OH coupling, followed by Fmoc deprotection and its analysis by EPR spectroscopy have been reported previously^7^. In the present study the TOAC spin label was inserted at position 32 and 15 in the Noxa sequence (Figs 1 and 6). Phosphorylation was accomplished by incorporation of Fmoc-Ser(PO(OBzl)OH)-OH for pSer13-Noxa. Noxa was characterized by mass spectrometry as shown previously^5^. *Electron Paramagnetic Resonance Spectroscopy data acquisition—*EPR spectra were acquired using a Bruker X-band (9.5 GHz) EleXsys E500 spectrometer equipped with the ER 4122 SHQ cavity. Temperature was maintained at 4 ± 0.2 °C with a quartz dewar insert and a nitrogen gas flow temperature controller. Spectra were acquired over a range of 120 G at varying microwave power. For all spectra, field modulation was at a frequency of 100 kHz with a peak-to-peak amplitude of 1.0 G. Typically, samples containing 8.0 nmol (50 μg) of Noxa, were resuspended in 20 μL of buffer. All EPR spectra were baseline-corrected and then normalized to the second integral.

### *In vitro* binding interaction assays

Unphosphorylated and phosphorylated 32-TOAC Noxa peptides were each incubated with purified, recombinant His-Mcl-1 in equimolar amounts at 4 °C with rotation in Dignam Buffer A (10 mM HEPES, 1.5 mM MgCl2, 10 mM KCl, 0.05% IGEPAL, 0.5 mM DTT, pH 7.9) for one hour. His-Mcl-1 was then immunoprecipitated with an anti-Mcl-1 antibody (S-19, rabbit IgG, Santa Cruz Biotech.), and the immune complexes were captured with protein G Sepharose beads. Proteins were resolved by SDS-PAGE, transferred to nitrocellulose membranes and immunoblotted for the presence of Mcl-1 (RC13, mouse IgG1, Santa Cruz Biotech.) and Noxa (114C307, mouse IgG1, Santa Cruz Biotech.).

### Molecular dynamics simulations

An unfolded state of human Noxa was generated, setting all backbone (ϕ,ψ) angles to (−135, 135). In order to produce a random structure of Noxa in solution, a 0.01 μs MD simulation of the protein in water was performed at a temperature of 400 K. The structure at the end of this simulation, with less than 20% in secondary structure content, was used for subsequent simulations of unphosphorylated^10^ and phosphorylated Noxa, as described below.

A 2.5 μs MD simulation of wild-type Noxa was performed in order to determine the structure of the unphosphorylated protein in solution. Since the structure of unphosphorylated Noxa converges to a hairpin structure in this time scale, we used this structure at the end of the trajectory to simulate the dynamics of Noxa phosphorylated at Ser13. Both unphosphorylated and pSer13-Noxa were solvated using TIP3P water molecules with a minimum margin of 25 Å between the protein and the edges of the periodic box. Na^+^ and Cl^−^ ions were added to the system to neutralize the charge of the system and to produce a NaCl concentration of approximately 150 mM. CHARMM 36 force field topologies were used for the protein, water and ions^17^,^18^.

MD simulations were performed using the program NAMD 2.9^19^. Periodic boundary conditions^20^, particle mesh Ewald^21^,^22^, a non-bonded cutoff of 9 Å and a 2 fs time step were used. The NPT ensemble was maintained with a Langevin thermostat (310 K) and a Langevin piston barostat (1 atm). The system was first subjected to energy minimization for 1000 steps, followed by a warming up period for 200 ps. This procedure was followed by equilibration for 0.01 ns with backbone atoms harmonically restrained using a force constant of 100.0 kcal mol^−1^ nm^−2^. Unrestrained production MD simulations of unphosphorylated and phosphorylated Noxa were continued for 2.5 μs each.

## Additional Information

**How to cite this article**: Karim, C. B. *et al.* Structural Mechanism for Regulation of Bcl-2 protein Noxa by phosphorylation. *Sci. Rep.* **5**, 14557; doi: 10.1038/srep14557 (2015).
