# Erratum: Structural Mechanism for Regulation of Bcl-2 protein Noxa by phosphorylation

**DOI:** 10.1038/srep15872

**Published:** 2015-10-29

**Authors:** Christine B. Karim, L. Michel Espinoza-Fonseca, Zachary M. James, Eric A. Hanse, Jeffrey S. Gaynes, David D. Thomas, Ameeta Kelekar

Scientific Reports 5: Article number: 14557; 10.1038/srep14557published online 09 28 2015; updated: 10 29 2015

The original HTML version of this Article incorrectly listed Ameeta Kelekar as being affiliated with ‘College of Biological Sciences, University of Minnesota, Minneapolis, MN 55455’. The correct affiliation is listed below:

Department of Laboratory Medicine and Pathology and Masonic Cancer Center, University of Minnesota, Minneapolis, MN 55455.

This has now been corrected.

